# Rehabilitation robots for the treatment of sensorimotor deficits: a neurophysiological perspective

**DOI:** 10.1186/s12984-018-0383-x

**Published:** 2018-06-05

**Authors:** Roger Gassert, Volker Dietz

**Affiliations:** 1 Department of Health Sciences and Technology, ETH Zurich, 8092 Zurich, Switzerland; 2 Spinal Cord Injury Center, Balgrist University Hospital, 8008 Zurich, Switzerland

**Keywords:** Robot-assisted therapy, Neurorehabilitation technology, Assist-as-needed, Stroke, Spinal cord injury, Locomotion, Upper limb function, Sensorimotor neurophysiology, Neuroplasticity

## Abstract

The past decades have seen rapid and vast developments of robots for the rehabilitation of sensorimotor deficits after damage to the central nervous system (CNS). Many of these innovations were technology-driven, limiting their clinical application and impact. Yet, rehabilitation robots should be designed on the basis of neurophysiological insights underlying normal and impaired sensorimotor functions, which requires interdisciplinary collaboration and background knowledge.

Recovery of sensorimotor function after CNS damage is based on the exploitation of neuroplasticity, with a focus on the rehabilitation of movements needed for self-independence. This requires a physiological limb muscle activation that can be achieved through functional arm/hand and leg movement exercises and the activation of appropriate peripheral receptors. Such considerations have already led to the development of innovative rehabilitation robots with advanced interaction control schemes and the use of integrated sensors to continuously monitor and adapt the support to the actual state of patients, but many challenges remain. For a positive impact on outcome of function, rehabilitation approaches should be based on neurophysiological and clinical insights, keeping in mind that recovery of function is limited. Consequently, the design of rehabilitation robots requires a combination of specialized engineering and neurophysiological knowledge. When appropriately applied, robot-assisted therapy can provide a number of advantages over conventional approaches, including a standardized training environment, adaptable support and the ability to increase therapy intensity and dose, while reducing the physical burden on therapists. Rehabilitation robots are thus an ideal means to complement conventional therapy in the clinic, and bear great potential for continued therapy and assistance at home using simpler devices.

This review summarizes the evolution of the field of rehabilitation robotics, as well as the current state of clinical evidence. It highlights fundamental neurophysiological factors influencing the recovery of sensorimotor function after a stroke or spinal cord injury, and discusses their implications for the development of effective rehabilitation robots. It thus provides insights on essential neurophysiological mechanisms to be considered for a successful development and clinical inclusion of robots in rehabilitation.

## Background

Rehabilitation robotics is a relatively young and rapidly growing field, with increasing penetration into the clinical environment [1]. In the late 1980s and early 90s a number of pioneering technological developments were launched, triggered by discoveries on training-induced recovery of sensorimotor function in animal models with damage to the central nervous system (CNS). The goal was to enhance the effects of functional training by providing increased therapy intensity and adaptive support in a controlled way.

The idea of using machines for rehabilitation dates back much earlier. In a 1910 patent, Theodor Büdingen proposed a ‘movement cure apparatus’, a machine driven by an electric motor to guide and support stepping movements in patients with heart disease. In the 1930s, Richard Scherb developed the ‘meridian’, a cable-driven apparatus to move joints for orthopaedic therapy. This human-powered mechanotherapy machine already supported multiple interaction modes, ranging from passive to active-assisted and active-resisted movements. A first robotic rehabilitation system was based on the concept of continuous passive motion (CPM), a stiff interaction mode in which the robot moves the joints along a predefined trajectory, independent of the contribution of the patient [2].

The first powered exoskeletons for therapeutic applications in SCI patients were introduced in the 1970s [3–5]. These systems used pneumatic, hydraulic, or electromagnetic (via cams and Bowden cables) actuators for position servocontrol. They included advanced features, such as actuated ankle flexion/extension, and hip adduction/abduction for increased stability [6] or the ability of a therapist to control the motion of the exoskeleton worn by the patient through his/her own movement (in a similar, connected exoskeleton) [7]. The first system for robot-assisted therapy of stroke survivors [8] was based on a stiff industrial manipulator and did not physically interact with patients, but rather moved a pad that patients had to touch to different locations.

A new era of neurorehabilitation robotics began in 1989 with the development of the MIT-MANUS [9], which was first tested clinically in 1994. Compared to industrial manipulators, this planar manipulandum presents inherently low mechanical output impedance (a frequency-dependent resistance to motion perceived at the interface between the human user and the robotic system) and provides unloading of the upper limb against gravity, thereby allowing to adapt support to the severity of the deficits. A few years later, force controlled devices for bimanual, cooperative grasping [10] and lifting [11] were introduced. This new generation of devices, using torque-controlled direct drive actuation, allowed for more advanced interaction control, ranging from passive movements for the most severely impaired patients, to active-assisted and active-resisted movements in moderately impaired patients. Furthermore, assistance could be automatically adapted to the patient’s performance. Around the same time, the Mirror Image Motion Enabler (MIME; [12]) was introduced, which supported paretic limb movements with a stiff industrial robot, controlled by the non-paretic limb by means of a motion digitizer (mirror-image therapy mode).

Developments of rehabilitation robots for the lower extremity began in 1994, with the design of the Lokomat [13], combining body-weight supported treadmill-training (BWSTT) with the assistance of a robotic gait orthosis. The Gait Trainer [14] realized a similar concept based on an end-effector design.

**Fig. 1:**
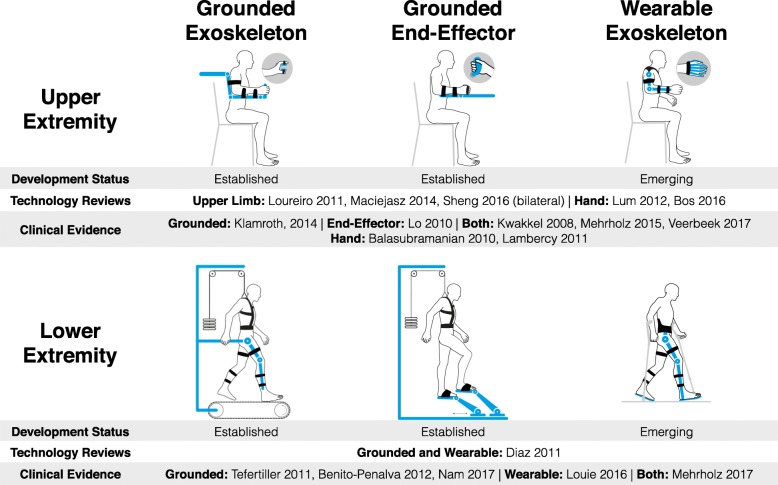
Schematic representation and classification of rehabilitation robots. Besides the extremity that is trained, rehabilitation robots can be broadly classified into grounded exoskeletons, end-effector devices and wearable exoskeletons. While the first two are well established, the latter are currently entering clinical application [17–21, 95, 122, 130–140]

The decades since these pioneering developments have seen an explosion of novel rehabilitation robots for both the upper and lower extremities, which can broadly be classified into grounded exoskeletons, grounded end-effector devices, and wearable exoskeletons (Fig. 1). These design approaches affect the level of control over the interaction (control of individual joints in exoskeleton devices vs control over selected joints or limb segments in grounded end-effector devices) as well as the output impedance of the device (resulting from the mechanical structure as well as actuator and transmission properties) and the ability to modulate this impedance through control. Grounded end-effector devices will typically achieve higher motion dynamics and allow the rendering of a wider range of impedances than exoskeleton devices with a serial kinematic structure, where proximal joints need to move distal joints. The latter requires large reduction ratios and results in high inertia and friction at the output where the patient is attached [15, 16]. These dynamics can only partially be compensated through control.

The number of new developments has been disproportionate to the penetration of these technologies into the clinical setting, likely due to the technology-driven approach of many engineering groups and the limited, albeit increasing, exchange of the field with therapists and clinicians. While a few randomized-controlled trials have confirmed efficacy of robot-assisted therapy equivalent to that of dose-matched conventional therapy [17–21], the majority of published devices were never clinically evaluated, or such an evaluation was limited to pilot studies on a few patients. Interestingly, many of these studies unsuccessfully aimed to demonstrate superiority of robot-assisted as compared to conventional therapy, despite the fact that there is currently no consensus on the optimal therapy program for an individual patient in the clinical field.

For a successful inclusion of robots in rehabilitation, fundamental knowledge about the physiological basis of the recovery of function is required. This knowledge is widely distributed and difficult for engineers to access and translate into design considerations. Consequently, in our opinion and experience, a close cooperation between engineers, therapists and clinical neurophysiologists/neurorehabilitation scientists is required from the very beginning of a development, and was shown to be successful in previous developments (e.g. of the Lokomat with the involvement of VD, a neurologist/clinical neurophysiologist [13]).

According to evidence from studies in cats [22], non-human primates [23], and humans [24], recovery of sensorimotor function after damage to the central nervous system (CNS) is based on the exploitation of neuroplasticity. It relies on physiological limb activation during the training of functional arm and hand movements, and the stimulation of appropriate peripheral receptors during automatically performed leg movements such as stepping. Rehabilitation robots should therefore enable and support such functional training.

This review aims to provide historical and clinical background of relevance to the field of rehabilitation robotics for engineers, basic and clinical neurophysiologists and therapists interested in and entering this exciting field. It introduces the neurophysiological basis for upper and lower limb functions that should be considered for the design of effective rehabilitation robots, and underlines the need for transdisciplinary collaborations for future developments. Before addressing aspects specific to upper and lower limb rehabilitation, general neurophysiological considerations of relevance for the design of rehabilitation robots will be discussed.

## Neurophysiological basis for the recovery of sensorimotor function after CNS damage

Stroke and spinal cord injury (SCI) are among the leading causes of adult long-term physical disability, with approximately 10 million people surviving a stroke and over 250′000 surviving a spinal cord injury (of which approximately 60% are incomplete) every year. Muscle weakness due to activation deficits represents the main disability following stroke and SCI, and frequently limits self-independence. Furthermore, following CNS damage, secondary effects such as spastic muscle tone (increased resistance to passive stretch) develop.

The aim of neurorehabilitation is to improve outcome of function after damage to the CNS, such as stroke and SCI, through intensive physical therapy. This goal is, however, difficult to define as the effects of conventional therapy can hardly be separated from the spontaneous recovery of function that occurs in parallel to the effects of the rehabilitative treatment [25]. In stroke [26] and SCI [27], most of the spontaneous recovery occurs within the first three months.

Therapy-induced recovery is mediated by neuroplasticity, and the goal of rehabilitation is thus to maximally exploit neuroplasticity in order to achieve an optimal outcome for the individual patient. However, neuroplasticity is limited, with most patients reaching a plateau after recovering approximately 70–80% of the initial impairment (stroke: [28–30]). Based on these studies it has been suggested that most of the observed recovery is spontaneous, without evidence for significant training effects on upper limb function. The recovery of neurological deficits is similar in young and elderly subjects, but the transfer into activities of daily living is reduced in the elderly [31]. As recovery is incomplete, compensatory movement strategies are also an important contributor to the mitigation of motor deficits [32], e.g. by enabling mobility through technical aids such as a wheelchair.

The recovery of function in persons with CNS lesion is much like a relearning process exploiting preserved sensorimotor circuits [33]. The relearning can be optimized by providing appropriate proprioceptive input to the spinal cord with the goal of maximally engaging preserved neural circuits. The extent of recovery depends on the severity of CNS damage and the individual neural capacity of a patient to regain a function. Cognition and motivation are important contributors to this relearning, especially for the upper limbs [34], and must therefore be considered during rehabilitation. ‘Normal’ movement performance can only rarely be restored after a stroke or SCI. Therefore, the goal of rehabilitation is not primarily to re-establish ‘normal’ movement patterns, but to enable ‘simpler’, less well-organized movements to achieve optimal outcome in mobility and independence during activities of daily living (ADL) for the individual patient [35].

There are basic differences in the recovery of upper and lower limb function. For instance, the exploitation of neuroplasticity is quite limited for arm and hand movements after a stroke, especially when the corticospinal tract is damaged. In addition, there are differences between cerebral and spinal cord damage. For example, the success of rehabilitation depends on the integrity of cognitive function, which is often impaired in post-stroke subjects.

Spasticity can contribute to the compensation of sensorimotor deficits [36–38], thereby assisting in the restoration of function. Spastic muscle tone can be used to partially compensate for the loss of limb activation in mobile patients. Consequently, movement generation takes place on a lower level of organization in the absence of cortical drive, i.e. spastic legs can provide body support during stance and gait in a stick-like manner [39]. However, this only holds for moderately affected, mobile patients, while in severely disabled patients, spastic signs such as muscle cramps may become exaggerated, requiring pharmaceutical interventions.

There is currently no consensus on the optimal therapy programs to promote recovery of motor function following CNS damage, and the understanding of recovery mechanisms is limited. Nevertheless, current evidence suggests that recovery requires active physical participation of patients during therapy [40]. Additionally, intensity (number of repetitions per unit of time) and dose (duration) of physical therapy are also thought to have a positive effect on outcome in both animal [22] and human [41–43] studies. These reports were challenged by a study showing no intensity effect and minimal gains in chronic post-stroke subjects [44]. This finding might be explained by the relatively low overall dose, ranging from 13.6 h to 26.3 h on the mean, whereas by applying a very high dose of 300 h, clinically meaningful gains were described [45]. This suggests that the doses provided in the standard of care are not sufficiently high, with implications for the further application of rehabilitation robots in the clinic and at home. Also, intensive task-specific multi-joint functional training does not necessarily improve performance in ADL [46] nor is it superior to single joint robotic training [47]. Nevertheless, there is some evidence for a transfer of task-specific training effects to untrained tasks [48].

Most of the factors that influence rehabilitation outcome are based on evidence from experiments in post-stroke patients as they represent a much larger patient group than patients with SCI. However, findings made in stroke concerning lower limb, i.e. stepping function, are usually also valid in SCI and can be transferred to this patient population. For example, the positive effect of training intensity on the outcome of ambulation in stroke subjects [41] could recently be confirmed for subjects with SCI [49]. For the upper extremity, hand function in SCI subjects is determined by the lesion level and the combined damage of central and peripheral nerval structures after a cervical injury [24]. In contrast, in post-stroke patients it greatly depends on the integrity of the corticospinal tract.

## General implications for robot-assisted therapy

The general neurophysiological considerations provide a strong basis for the application of robots in rehabilitation. Robot-assisted rehabilitation provides a standardized environment, in which both therapy intensity and dose can be increased. In a conventional setting, hemiparetic patients typically perform about 30 movement repetitions with their affected upper limb in a 45-min session [50], whereas robot-assisted therapy has achieved over 1000 repetitions per session [18]. Active physical and cognitive engagement of patients during therapy are crucial for recovery. This can be promoted through adaptive assistance [51], in a way to avoid slacking of the patient [52], as well as through cognitive challenge [53], automated task difficulty adaptation [54, 55] and motivating feedback [34]. Feedback about movement performance can not only enhance motivation but also facilitate plasticity in the motor cortex if it arrives synchronously with motor output [56].

**Fig. 2:**
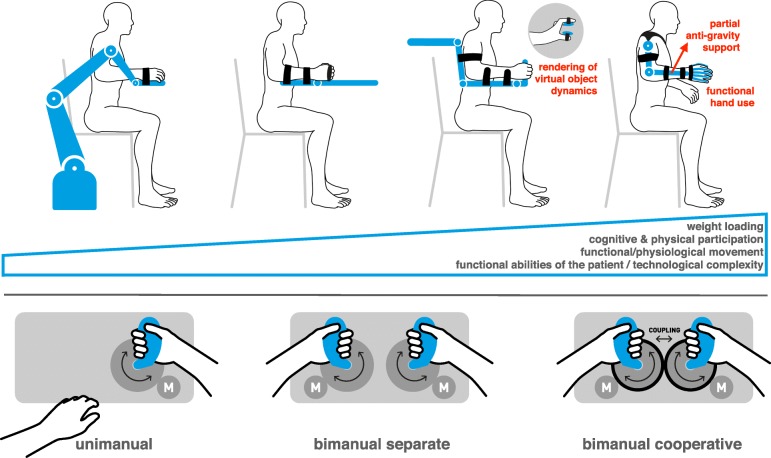
Upper panel: Evolution of upper extremity rehabilitation robots. From stiff (high impedance) industrial manipulators to dedicated rehabilitation robots providing control at the distal effector or over each joint, including the rendering of virtual object dynamics resulting in somatosensory feedback. Further evolution of the technology will see wearable systems providing support not only during therapy sessions, but also during activities of daily living in the home environment, allowing physical interaction with real objects. Lower panel: Task-specific design of hand rehabilitation robots. Functional hand movement training should focus not only on unimanual, i.e. reach and grasp tasks (left), but should also include bimanual separate tasks (middle), as well as cooperative movement tasks that are employed, e.g., when opening a bottle (right)

**Fig. 3:**
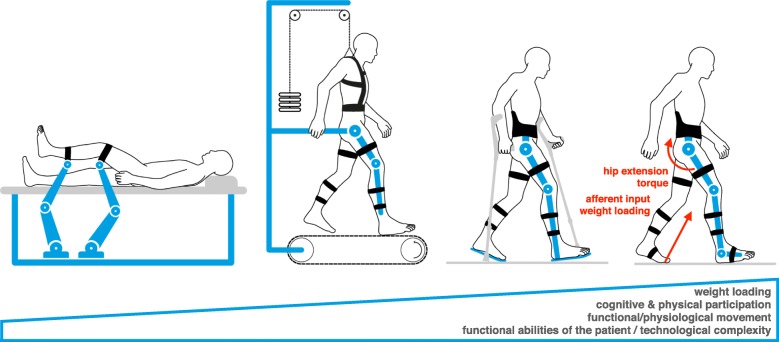
Evolution of lower extremity rehabilitation robots. Since their introduction, rehabilitation robots for the lower extremity have evolved from stiff industrial robot arms to guide the limb passively, without cognitive or physical involvement of the patient, to systems allowing for active engagement of patients through adapted support and body weight unloading in a vertical posture. Currently, wearable exoskeletons are being introduced into clinical practice, promoting even more active engagement of the patient, while balance is provided by crutches. Future exoskeletons will support balance to the degree needed. The three systems to the right are inspired by neurophysiological insights, stimulating afferent receptors through, e.g., weight loading, ground contact and assisted hip extension to trigger leg flexion movements. From left to right, patients require increasing functional abilities, while the robotic systems provide less support. Most patients will benefit from several of these systems (from left to right) during different phases of recovery

Severely affected patients can benefit from passive/highly-assisted movements and gravity support by exoskeletons that provide control over all relevant joints (Figs. 2 and 3). In order to minimally interfere with and alter functional movements in less affected patients [16], nor influence automated assessments based on integrated sensing [57], rehabilitation robots should have low inherent impedance [58], or require the ability to adapt output impedance through control [16]. This requires a careful selection of kinematic structure, actuation/transmission and integrated sensing based on the functional tasks to be trained, the targeted patient population and the severity of sensorimotor deficits.

## Rehabilitation of arm/hand function

The versatility and complexity of arm and hand movements with unique functions such as unimanual reaching, grasping and manipulation, as well as bimanual separate and cooperative movements, differ fundamentally from stepping movements with a more automatic movement control. Skilled hand and finger movements reflect cultural achievements in the evolution [59] that are associated with a specific cortico-motoneuronal control [60], i.e. direct projections from the cortex to motoneurons in the spinal cord which innervate arm/hand muscles. As a result, arm, and especially distal hand function are often severely impaired following CNS damage, greatly limiting patients in their ability to perform ADL [61]. The severity of impairment and, consequently, any recovery of function is related to the extent of damage of the corticospinal system [62, 63]. Functional training approaches and, consequently, devices supporting unilateral arm and hand movements [64, 65] should thus be directed towards the abilities patients require for ADL, i.e., most importantly unimanual and bimanual reach and grasp tasks [66]. Furthermore compensatory approaches and assistive devices have to be considered for more severely impaired patients.

### Neurophysiological factors influencing the recovery of upper limb function

In general, the recovery of arm/hand function following CNS damage is limited when compared to gait in post-stroke [41] and cervical spinal cord injured [67] subjects, even if intensive therapy is applied. In patients with a cervical SCI, arm function depends on the level of the lesion. An injury level at C5/C6 results in a ‘passive’ hand function (supination movement at the elbow joint for hand opening) or, frequently, at C6/7, in a tenodesis grasp. This grasp is defined as a hand function when some forearm extensor muscle activation is preserved [68]. It allows to close the hand by wrist extension movements with the fingers in a slightly flexed contracture position. Some spastic muscle tone is required to perform such simple grasp movements [24].

In post-stroke subjects, outcome of upper limb function critically depends on the integrity of the corticospinal tract (CST) [63, 69]. A stroke with damage to the CST results in lasting impairment of hand and finger function and an unbalanced muscle tone with forearm flexor hypertonia and extensor weakness that contributes to the inability to perform finger extension and hand opening movements [60]. These patients also suffer from difficulties in the grasping and manipulation of objects, while some proximal arm function is usually preserved. Most reports show that in patients with damage to the CST, even with intensive rehabilitation measures, little recovery [28, 30], particularly of hand and finger function [70], can be expected.

In contrast, the recovery in patients with an intact CST is proportional to the initial impairment, with patients recovering approximately 70–80% of the initial impairment (proportional recovery rule) [28–30]. Some studies indicate that training effects in these patients are small or absent [46], i.e. only a minor dose-response effects occur [44]. However, there is also evidence that a higher dose of practice, especially when applied early after a stroke, leads to a better outcome of motor function of the paretic arm [41, 43, 71].

Early after stroke flaccid arm muscle paresis prevails, i.e. the limbs are weak and do not resist passive displacement. With the development of some spastic muscle tone, needed to perform rudimentary grips, the training of residual muscle function can be initiated [24]. In this stage, the focus of therapy/training should be directed to enable the execution of simple reach and grasp movements. In the weeks following stroke, spastic muscle tone usually becomes more pronounced in the forearm flexor than in the extensor muscles, as the antigravity muscles have more muscle mass [39, 72]. This can again impair the execution of functional reach and grasp movements. However, some spastic muscle tone in the forearm muscles allows the performance of a tenodesis grasp, which is important for the execution of ADL, not only in SCI but also in post-stroke subjects.

Patients typically compensate for their sensorimotor deficits through the involvement of the non-paretic arm/hand, leading to learned non-use of the paretic arm [64, 73]. Therefore, one important approach to rehabilitate hand function after stroke was presented in the form of constraint-induced movement therapy (CIMT). This was based on the idea of enhancing recovery of function by reducing interhemispheric inhibition of the stroke hemisphere [74]. By immobilizing the non-affected hand the patient is forced to use the paretic hand/arm for the performance of ADL [64]. However so far, a superior effect of CIMT compared to other therapy approaches was not reported [75].

During the course of upper limb rehabilitation, the support provided should always be kept to a minimum in order to make the training challenging with a maximum of individual effort and contribution to movement performance by the patient (for review [24]). However, the optimal level of assistance also depends on the severity of impairment [70]. Most stroke patients will benefit from gravity support, allowing them to perform functional movements by their own effort [76]. Without such support, shoulder abduction, which is important for object manipulation, may limit elbow extension and result in concurrent elbow, wrist and finger flexion, i.e. so-called flexion synergies after stroke [77]. This can affect the execution of functional hand movements.

Many upper limb movements involve the use of both hands. However, only a few studies provide a neurophysiological basis for the training of bimanual movements [78]. Bimanual training of reaching and grasping tasks in stroke patients has been suggested to be more effective in improving unilateral execution of these tasks with the affected arm than unilateral training alone [79]. This might be a result of stronger recruitment of the contralesional hemisphere through bilateral compared to unilateral training [80]. However, there is currently no clear evidence that bimanual training is superior to CIMT [65, 81, 82], or unconstrained unimanual training [83].

The involvement of the unaffected hemisphere in movement control of the paretic hand might be even stronger in a special type of bimanual movement, where one hand supports the action of the other one by generating equal but opposed forces/torques, e.g. when opening a bottle or cutting bread. Such cooperative hand movements are based on a task-specific control: a ‘neural coupling’ of the hemispheres, i.e. both ipsi- and contralateral hemispheres become involved in the control of each of the two hands during cooperative hand movements [84]. Consequently, in post-stroke patients during the training of cooperative hand tasks, the unaffected hemisphere supports movements of the paretic hand and arm [85]. However, the effect of a cooperative training on the outcome of hand function remains to be determined.

Finally, while the recovery of finger function is limited, basic functions such as opening and closing the hand should also be trained, as most of the interaction with the environment during ADL involves grasping and releasing objects. Besides motor function, somatosensory function is also of importance during object grasping: shaping and maintaining a stable grasp during the manipulation of an object relies on the processing of somatosensory input, determined by the mechanical properties of the manipulated object [86]. Somatosensory function is often impaired after CNS damage, leading to a visual compensation of movement control. However, in some patients it can recover spontaneously or through dedicated training [87].

### Implications for robot-assisted therapy of upper limb function

The combination of kinematic complexity and functional impairment makes the design of robotic devices to train arm, hand and finger function after CNS damage particularly challenging. Following the initial developments based on stiff industrial manipulators, end-effector-based devices for planar (MIT-MANUS; [88]) and 3D (Gentle/S, [89]) reaching movements were introduced to allow more active contribution of the patient while limiting the apparent impedance of the robot. Subsequent developments focused on incorporating additional degrees of freedom (DOF) related to wrist [90] and hand opening/closing function (Gentle/G [91]). For the functional training of three-dimensional arm movements with guidance at the three proximal joints, ARMin, a grounded, powered exoskeleton was developed, which also integrates grasp and release function [92, 93] (Fig. 2, upper panel).

Independent of their kinematic configuration, all of these systems can partially or fully unload the arm against gravity. This approach reduces the effect of flexor synergies, and allows the performance of hand movements within a larger workspace. However, the complex structure and geared actuators of such devices with their reflected inertia limit the interaction quality and the ability to adapt the level of support [16]. The large output impedance may render the active initiation of movements more difficult, and potentially alter natural movement dynamics. Therefore, a trade-off between the number of DOF and the quality of the physical interaction exists, limiting the application of these devices to specific stages of recovery. For example, training with a powered whole-arm exoskeleton is mainly indicated for stroke subjects with severe arm paresis early after the incident. Similar effects can also be achieved by using passive devices for gravity support to the upper limb, to enable self-initiated movements [94].

Robot-assisted approaches should also consider the training of bimanual and cooperative movement tasks as they are important during ADL (Fig. 2, lower panel). Bimanual training was a focus of some early studies [10], but its potential has not been sufficiently explored and deserves further investigation. Many upper extremity systems developed and clinically evaluated so far could also be used for bimanual training, by combining two devices in a mirrored configuration. The training of cooperative hand movements (e.g. opening a bottle) has been proposed using a dedicated device [84], and can also be achieved by virtually coupling two unimanual devices through control.

Due to the biomechanical and neural complexity of hand and finger movements, robot-assisted rehabilitation of hand and finger function became a focus only recently. Most rehabilitation robots for hand function have been based on end-effector designs, used either independently or in combination with grounded exoskeletons or end-effector type arm devices (Fig. 2). Several groups have also made attempts to develop exoskeleton systems for the hand, some of which assist independent finger motion, generally resulting in highly complex devices that underwent none to little clinical evaluation. A review [19] found that only 25% of 30 hand rehabilitation robots had been clinically tested, and many devices had been considered too complex for clinical use. However, such complexity might not be necessary when the focus is directed to the basic function of opening and closing the hand [95]. This might be sufficient given the limited potential for the recovery of finger function following CNS damage, while remaining highly relevant for ADL. Finally, hand opening/closing can also be supported through wearable assistive technology, such as soft robotic gloves [96, 97], which could be worn during the performance of ADL.

Interaction with the environment occurs mainly through the hands and generates somatosensory feedback. However, somatosensory function is often impaired after CNS damage. Therefore, neurorehabilitation devices for the upper extremity should train hand and, as far as possible, finger function, providing both visual and haptic feedback [53]. Training should include tasks which are functionally relevant for ADL, such as grasping and releasing objects with rendered virtual dynamics to also train somatosensory function and sensorimotor integration [98]. Finally, most upper limb training devices are embedded in computer games to reflect the cognitive nature of these tasks and motivate patients. In a meta-analysis, the application of virtual reality (VR) games was found to be potentially useful for the improvement of arm function after stroke [34].

In conclusion, a good, mainly spontaneous, recovery of upper limb function after a stroke can be expected when the integrity of the CST is preserved. There is some evidence that higher dose of practice leads to improved function, especially early after stroke. Nevertheless, in cases with damaged CST the recovery is limited and neither depends on the approach nor on the dose of training. Unimanual robot-assisted therapy approaches should be complemented by bimanual (cooperative) approaches. These should also incorporate the training of basic hand function and interaction with virtual object dynamics that generate somatosensory feedback. In the future, it will be possible to at least partially compensate for remaining deficits with wearable assistive robotics.

## Rehabilitation of locomotor function

Locomotor movements are performed more automatically than arm and hand movements. Corticospinal control mainly serves the goal to voluntarily alter the stepping rhythm, e.g. to correct the stepping direction or amplitude to overcome obstacles. Accordingly, corticospinal projections to lower limb motoneurons in humans are stronger to the flexor than to the extensor muscles [99, 100]. The rehabilitation of locomotor function is simpler than that of upper extremity function, and basic mobility can usually be restored in post-stroke subjects by using the paretic limb as a stick to support the body [24].

Passive orthoses can assist foot dorsiflexion in the swing phase of stepping. In SCI subjects, some proximal leg muscle activation is required for a successful locomotor training [101]. Besides this, the rehabilitation of locomotor function in post-stroke and SCI subjects is similar. In severely affected subjects, mobility can be restored with a wheelchair or other mobility aids. Nevertheless, the primary goal of rehabilitation is to restore sufficient lower-limb function for patients to ambulate without walking aids.

### Neurophysiological factors influencing the recovery of locomotor function

Thirty years ago, rehabilitation after CNS damage was focused on the strengthening of leg muscles to a level where patients were able to perform stepping movements on parallel bars with the support of their arms [102]. In the early nineties, functional locomotor training with body unloading of para−/tetraparetic SCI subjects was introduced. This was based on the observation that locomotor function in cat SCI models recovers quite well during treadmill training with body-weight unloading (body weight supported treadmill training, BWSTT) [103]. In incompletely paralyzed SCI patients, BWSTT has been shown to result in a similar outcome of stepping function compared to a conventional rehabilitation approach [104]. In post-stroke subjects no gain in outcome was found during BWSTT compared to an unspecific physical exercise program [105]. The BWSTT training of SCI subjects is physically demanding and requires two physiotherapists who assist leg movements from both sides. As a result, training time is limited to about half an hour per day, even though many patients would tolerate more therapy. Yet, such a dose increase has been associated with a better outcome [41].

Movement speed during locomotor training represents another factor that influences outcome. In ambulatory stroke patients, a successive increase (according to principles of sports physiology) of treadmill speed after a 4-week training period resulted in a better walking ability than conventional gait training [106]. Furthermore, locomotor training was shown to be most efficient when delivered in a real-world environment [107].

In severely paralyzed patients with an SCI, automatic stepping movements can be induced, associated with a physiological leg muscle activation (i.e. close-to normal timing of electromyography (EMG) patterns with reduced amplitude), when patients stand on a moving treadmill with the body unloaded up to 80% [108, 109]. This leg muscle activation is triggered by load receptor input from contact forces during the stance phase of gait [110]. Such a physiological limb muscle activation was found to be the prerequisite for positive training effects and improvement of locomotor function in rodents [22] and patients with a stroke or SCI (for review [24]). With the onset of voluntary control of some proximal leg muscles, body unloading can be reduced and self-induced stepping movements become possible. This is associated with an increase in strength of leg muscle activation. Thus, during the course of training, body un−/reloading has to be adapted to the actual degree of paresis.

Most of the recovery of function occurs during the first three months after CNS damage. However, also in chronic patients with an incomplete SCI and stroke a significant gain in gait velocity, endurance, and performance can be achieved by an automated locomotor training [102]. Further improvement of locomotor function after damage to the CNS is associated with only minor changes in the leg muscle activity pattern, and relies more on a better coordination between the legs and an adapted spastic muscle tone (stroke: [111]; SCI: [37]).

Hip extension at the end of the stance phase is an essential stimulus for the leg muscle activation during locomotion, especially for initiating the stance to swing transition with an appropriate change in leg muscle activation [110]. This is in line with cat experiments, where flexor bursts were automatically generated at the end hip extension despite complete SCI [112, 113]. In robot-assisted gait training systems, leg flexion movements are usually imposed by the robot, leaving the subject passive. Over time, this leads to a rarefaction [114] and dysfunction [115] of leg flexor motoneurons, i.e. the peripheral nervous system, deprived of supraspinal input, undergoes degenerative changes. In completely paralyzed patients with an SCI who do not undergo a functional locomotor training, spinal neuronal circuits underlying stepping movements become silent even when appropriate proprioceptive input is provided. On a longer term this results in a neuronal dysfunction below the level of the lesion in both rodents [116] and patients with SCI [117].

Today we know that bipeds use a quadrupedal coordination during locomotion, i.e. arm movements represent an integral part of locomotion [118, 119] and, therefore, might be included in locomotor training programs. In fact, recent experiments indicate that arm movements induce an increase in leg muscle activity during stepping [120].

### Implications for robot-assisted therapy of lower limb function

Functional gait training positively affects the recovery of locomotor function, but is personnel-intensive and physically demanding for the therapists. This situation triggered the development of robotic devices to assist leg movements during stepping on a treadmill with the body partially unloaded [13]. Robot-assisted BWSTT has been shown to be as effective as overground stepping with the support of physiotherapists [121]. Together with the fact that training intensity has a positive effect on the recovery of locomotor ability in post-stroke [41] and SCI [49] subjects, this motivates the use of robot-assisted BWSTT, allowing longer training times (and thus higher dose) with less personnel. Furthermore, this approach provides a standardized training environment and allows an objective assessment of the changes achieved during the course of rehabilitation [57]. A systematic review that examined the effect of electromechanical and robot-assisted gait training in post-stroke subjects showed that patients receiving such training are more likely to achieve independent walking than people who received gait training without such devices [122].

During the course of rehabilitation, the physical support has to be continuously adapted to the actual needs of the patient, with the objective of maximizing active participation of the patient by reducing and selectively providing assistance [24]. With the recovery of locomotor function (especially of proximal joints), a transition from a grounded exoskeleton to a grounded end-effector device can take place (e.g. [123]). This can be followed by the execution of stepping movements on normal ground with reduced support from a wearable robotic exoskeleton or mobility aid.

Initial developments for robot-assisted gait training were focused on patients with almost complete paralysis as a result of SCI, where training is most demanding for therapists. These patients can hardly actively contribute to the leg movements. Therefore, high assistive torques are required, typically resulting in robotic devices with high output impedance. With increased penetration of this technology into clinics, locomotor training using such devices was expanded to patients with a stroke or incomplete SCI, who require less and/or asymmetric assistance. However, this is difficult to achieve by devices with a high output impedance, as these behave more like a velocity than a torque source. Consequently, novel control approaches [124] and devices with low intrinsic impedance [125] were developed to better adapt the physical support to these patient populations. These efforts need to be continued, also to assure that automated assessments reflect the current impairment level of a patient, and are not masked by the device dynamics [57].

The field of lower-extremity robot-assisted therapy is progressing towards wearable powered exoskeletons (Fig. 3). These combine the advantages of grounded devices with the ability to train in a real-world environment and provide higher levels of subject participation and challenge [58]. Even more than in the case of grounded devices, it is a challenge to achieve low output impedance together with the provision of sufficient assistive torque in wearable exoskeletons, as all links and joints are integrated and thus carried by the moving exoskeleton. This results in weight and complexity constraints that limit both the number of DOF that can be actuated and the transparency (ability of the system to get out of the way) that can be achieved. The high reduction ratios required to generate sufficient torque increase the output impedance of the device, thus limiting the capacity to adapt the support to changing abilities of the patient. With technological progress, it might become possible to modulate the output impedance of each joint. Through this approach, hip extension might be enforced to trigger physiological leg flexion movements for the initiation of the swing phase (cf. [110]), which the exoskeleton could passively follow. Such an approach would allow a better adaptation of the support to the individual patient, enable more dynamic motion, and prevent degenerative changes in the peripheral nervous system.

With the ability to partially support balance with wearable exoskeletons, the hands become free from holding crutches. This will facilitate arm swing, which represents an integral part of locomotion (for review see [118]). These devices will not only allow to continue therapy after hospital rehabilitation, but also to compensate for remaining deficits by providing appropriate assistance in ADL.

In conclusion, in post-stroke patients training leads to a good recovery of stepping function using spastic muscle tone for body support. In SCI, some remaining proximal leg muscle function is required to allow a successful training and recovery of function. The activation of load (re-loading of the body as far as possible) and hip-joint related (hip extension) receptors leads to a physiological leg muscle activity pattern during stepping and, consequently, to dose-dependent training effects. Accordingly, devices are required which can adapt the support and impedance of individual joints according to patients’ impairment. The development of wearable robotic gait orthoses with integrated balance support will further promote functional training, engagement and motivation, and lead to systems that can provide long-term assistance in the home environment (Fig. 3).

## Conclusions

Rehabilitation training of the upper and lower limb should be founded on neurophysiological insights, independent of whether it is performed conventionally, or with the support of robotic devices. After CNS damage, improvement of sensorimotor functions occurs to a large degree spontaneously and can further be achieved by an exploitation of neuroplasticity. This is reflected in a physiological limb muscle activation that might serve as a marker for the achievement of training effects. This requires voluntarily performed upper limb movements, or an activation of appropriate receptors for a purposeful activation of lower limb muscles, i.e. during stepping movements.

The potential for a recovery of function differs not only between upper and lower limbs, but also between neurological disorders such as stroke and SCI, and requires the development of technology accounting for these differences. Table 1 summarizes the main aspects of neurorehabilitation and outcome in these disorders, as well as the implications for rehabilitation technology.

**Table 1:** Main aspects of neurorehabilitation and outcome, and their implications for rehabilitation technology

Limb	Condition		Typical recovery course	Goal	Rehabilitation approach	Technology
**UL**	stroke	damaged CST	little recovery, esp. chronic impairment of hand/finger extension	prox. arm muscle activation; avoidance of muscle contractures; use of impaired limb for support/holding function	prox. arm muscle strengthening; continuous passive limb motion; training of compensatory strategies	therapy: passive mobilization (position control) or weight support for self-initiated proximal movements; active/passive hand module with extension bias assistance: supported arm/hand motion (admittance control) vial intention detection (e.g. force, EMG, gaze)
		intact CST	spontaneous recovery of approx. 70–80% of intial arm/hand impairment	arm reaching and simple grasping function; uni−/bimanual ADL functions	functional reach/grasp and bimanual (cooperative) hand movements; strengthening of wrist/finger extensors; simple movement training with transfer to ADL; limited dose-dependent training effects: subacute > chronic stage	therapy: proximal gravity support during reach/grasp; training of individual joints using dedicated devices, including hand/fingers, as well as (cooperative) bimanual training (Fig. 2) assistance: passive proximal gravity support combined with active wrist/finger support via residual function amplification (force/EMG control)
	SCI (incomplete)	typical lesion level C6/7	spastic forearm flexor muscle tone impeding the development of tenodesis grasp	tenodesis grasp; bimanual grasp		assistance: active exoskeleton/glove to facilitate wrist and finger flexion/extension triggered by proximal arm motion (e.g. joint angle sensor)
**LL**	stroke	hemiparesis	spontaneous recovery; spastic muscle support; reduced level of stepping movement ability	non-assisted ambulation	generation of appropriate afferent input from load (body un/reloading) and hip receptors (hip extension) during stepping; importance of stepping velocity and hip extension (initiation of swing); dose-dependent training effects	therapy: body-weight support according to paresis; adapted movement support (position/admittance control for severe impairment and variable impedance control for mild/moderate impairment; Fig. 3); leg flexor activation through robotically assisted hip extension
	SCI (incomplete)	paraparesis	some prox. leg muscle function and spastic muscle tone required for stepping ability	assisted/independent ambulation		

*UL* upper limb, *LL* lower limb, *SCI* spinal cord injury, *CST* corticospinal tract, *ADL* activities of daily living, *EMG* electromyography

Training effects in upper compared to lower limbs are limited and are mainly determined by corticospinal tract integrity. Nevertheless, intensive, highly dosed training has beneficial effects, especially early after stroke. Training devices should unload (and gradually re-load) arm movements against gravity, incorporate hand function for reach and grasp training and use the motivating and cognitively engaging effects of virtual reality, with a focus on the ADL that are most important to the individual.

For the lower limbs, the effects of training on the recovery of sensorimotor function seem to depend on both their intensity and dose. Rehabilitation robots are ideal tools to complement classical functional therapy by allowing a standardized and intensive training with individually and continuously adapted physical support. Grounded exoskeletons and end-effector devices as well as wearable exoskeletons seem to be equally effective in the improvement of function. However, their suitability depends on the phase of recovery, and the individual impairment (Fig. 3).

Rehabilitation robots should always provide targeted physical support adapted to the functional abilities of the patient in a way to enable functional movements. This has strong implications for the design, instrumentation and control of such systems. These should be able to adapt their output impedance and physical support to the actual state of the patient and the task at hand, without altering functional movement patterns through their apparent dynamics. Patients will likely train with different devices throughout the recovery phase during rehabilitation, to optimally adapt movement complexity and physical support to the current state and functional abilities of the patient (e.g. transitioning from left to right in Fig. 3). To deal with this challenge, the design of future robotic rehabilitation systems should also consider the relevance of particular joints during functional movement (e.g. the hip joint plays a larger role in locomotion than the knee and ankle joints; [110]) and their potential for recovery (e.g. limited recovery of individual finger movement).

There are currently a number of novel and exciting developments in and around the fields of rehabilitation engineering and rehabilitation sciences. Advances in material sciences will allow lighter, more customizable structures with more tightly integrated actuation and sensing. Furthermore, there is an increasing focus on combining robotics with non-invasive [126, 127] and invasive [128] brain-machine interfaces or neuroprosthetics, with the aim of promoting independence during activities of daily living. These approaches are at an early stage and still face a number of challenges. Nevertheless, even an optimal exploitation of neuroplasticity (cf. [24]) will not result in a full functional recovery. The field should therefore focus on wearable systems that not only support functional therapy, but that can also serve as assistive devices to compensate for persisting sensorimotor deficits. Advanced actuation, sensing and control approaches will make these systems more robust and applicable for ADL tasks, and applicable both in the clinic and at home. In the future, it can be expected that wearable devices continuously adapt and reduce support until recovery plateaus, and then compensate for chronic impairments.

A number of further challenges remain for the field of robot-assisted therapy and assistance, many of which will also require collaboration with industry and government bodies. Adoption of robotic devices is driven by cost and reimbursement systems, and research should therefore also focus on identifying the simplest, most effective technical solutions that can support the rehabilitation process.

Finally, future rehabilitation approaches will not only profit from the inclusion of robots, but also from an advanced understanding of neurophysiological mechanisms underlying normal and impaired sensorimotor functions, enabled by the use of robots as scientific tools [129]. Resulting insights will benefit the development of advanced rehabilitation robots, and further promote collaboration between engineers, therapists and clinical neurophysiologists.
